# Genetic Approaches for Sports Performance: How Far Away Are We?

**DOI:** 10.1007/s40279-019-01164-z

**Published:** 2019-11-06

**Authors:** Michael J. Joyner

**Affiliations:** Department of Anesthesiology and Perioperative Medicine, Mayo Clinic, Rochester, MN 55905 USA

## Abstract

Humans vary in their ‘natural ability’ related to sports performance. One facet of natural ability reflects so-called intrinsic ability or the ability to do well with minimal training. A second facet of natural ability is how rapidly an individual adapts to training; this is termed trainability. A third facet is the upper limit achievable after years of prolonged intense training; this represents both intrinsic ability and also trainability. There are other features of natural ability to consider, for example body size, because some events, sports, or positions favor participants of different sizes. In this context, the physiological determinants of elite endurance performance, especially running and cycling, are well known and can be used as a template to discuss these general issues. The key determinants of endurance performance include maximal oxygen uptake $(\dot{V}{\text{O}}_{2\hbox{max} } )$, the lactate threshold, and running economy (efficiency in the case of cycling or other sports). In this article, I use these physiological determinants to explore what is known about the genetics of endurance performance. My main conclusion is that at this time there are very few, if any, obvious relationships between these key physiological determinants of performance and DNA sequence variation. Several potential reasons for this lack of relationship will be discussed.

## Key Points

**Table:** 

‘Natural ability’ or talent is a widely appreciated feature of many elements of sports performance.
The assumption is that key physiological elements of talent are embedded in, or explained by, interindividual differences in DNA sequence.
At this time, interindividual differences in DNA sequence explain only a small fraction of the physiology underpinning sports performance.

## Introduction

Over the past 50 or so years, the key physiological determinants of endurance exercise performance have emerged. These include maximal oxygen uptake $(\dot{V}{\text{O}}_{2\hbox{max} } )$, the lactate threshold, and efficiency. In the case of distance running, efficiency is typically referred to as running economy because it is difficult to calculate efficiency in a strict engineering context in running humans [1]. By contrast, it is much easier for cycling.

Data on these three variables can be modeled to predict performance, and there are field tests that incorporate several of these variables that are also highly predictive of performance. For example, in the early 1990s I took emerging evidence that humans run the marathon at a pace similar to their running speed at lactate threshold, and calculated a theoretical upper limit, at least at that time, for the ‘fastest’ potential marathon performance by men [2]. This model also reasonably predicted the performance of a given individual. Likewise, so-called velocity at $\dot{V}{\text{O}}_{2\hbox{max} }$ was shown to be highly correlated with running performance [3]. This latter measure incorporates both $\dot{V}{\text{O}}_{2\hbox{max} }$ and running economy into one metric.

The basic idea underpinning these factors is that they interact in a predictable way. $\dot{V}{\text{O}}_{2\hbox{max} }$ can be seen as the upper limit of aerobic capacity, the lactate threshold related to the fraction of $\dot{V}{\text{O}}_{2\hbox{max} }$ that can be sustained for a duration longer than a few minutes, and efficiency or economy related to the actual power output or speed during a race that can be generated at a given V̇O_2_. Additionally, the physiological determinants of $\dot{V}{\text{O}}_{2\hbox{max} }$ and the lactate threshold are well understood. Less is known about the physiological determinants of efficiency/economy. The question then is, if the physiological determinants of $\dot{V}{\text{O}}_{2\hbox{max} }$ and the lactate threshold are well understood, what is known about the contribution of DNA variation to these factors?

Before I go on, I want to share two sets of assumptions related to the physiology behind $\dot{V}{\text{O}}_{2\hbox{max} }$ and the lactate threshold. First, for $\dot{V}{\text{O}}_{2\hbox{max} }$, the primary physiological determinants under most circumstances in most humans are related to maximum cardiac output and stroke volume, along with red cell mass or total body hemoglobin [4]. In other words, the ability of the heart to pump large quantities of oxygenated blood to the contracting skeletal muscles is absolutely critical. While this is not true in every case and in every circumstance, for example chronic obstructive pulmonary disease (COPD), where the lungs can become limiting, it is true for the vast majority of situations. Second, the lactate threshold reflects, in large part, some combination of skeletal muscle mitochondrial content and function in the contracting skeletal muscles and perhaps capillary density [5, 6]. Efficiency/economy is much more complex and likely sport-specific. It also has an element of the competitive medium that needs to be considered. Examples include wind resistance during high-speed cycling versus lower-speed running, or water resistance for sports such as swimming or rowing [1].

Therefore, with this general perspective as a background, I will next try to ask what is known about the genetic contributions to the major physiological determinants of endurance exercise performance. A key question then is what constitutes ‘genetic’. One approach is to focus on the heritability of key traits related to athletic performance. These are typically statistical arguments based on the correlation of a given trait between family members, most notably mono- or dizygotic twins. If the correlation between monozygotic twins is greater than the correlation between dizygotic twins then the interpretation is that this similarity is due primarily to greater similarities in the DNA of monozygotic twins than dizygotic twins [7]. For $\dot{V}{\text{O}}_{2\hbox{max} }$, the heritability can be very high for monozygotic twins, consistent with the idea that there is a major genetic component to this variable. Twin (and other family) studies also indicate that there is a significant genetic component to the increase in $\dot{V}{\text{O}}_{2\hbox{max} }$ seen with a few months of fitness-type training [8, 9].

While the observations highlighted above suggest there is a strong genetic component to training, specific DNA variants associated with $\dot{V}{\text{O}}_{2\hbox{max} }$ and how $\dot{V}{\text{O}}_{2\hbox{max} }$ responds to training have been hard to find. While a number of small effect size variants considered in concert seem related to the rise in $\dot{V}{\text{O}}_{2\hbox{max} }$ with training, no variants alone or in combination that are clearly linked to canonical biological pathways likely to underpin cardiac output and red cell mass have been identified [10–13].

The issue of limits of genetic ‘causation’ is also part of a general trend in genomic research for complex human traits that has accelerated in recent years following the completion of the human genome project. In the late 1990s and early 2000s, it was generally assumed that a limited number of gene variants would explain much of the risk of developing common non-communicable diseases. The idea was that once these variants were identified, a host of new approaches to diagnosis, prevention, and therapy would emerge. Unfortunately, this vision has not been realized and hundreds of gene variants with small effect sizes have been associated with complex non-communicable diseases. Importantly, their role in the diagnosis, prevention, and therapy for these diseases remains obscure. These larger issues related to genomics and complex disease-related traits have been discussed in detail elsewhere [14].

## Oxygen Transport Cascade

**Fig. 1:**
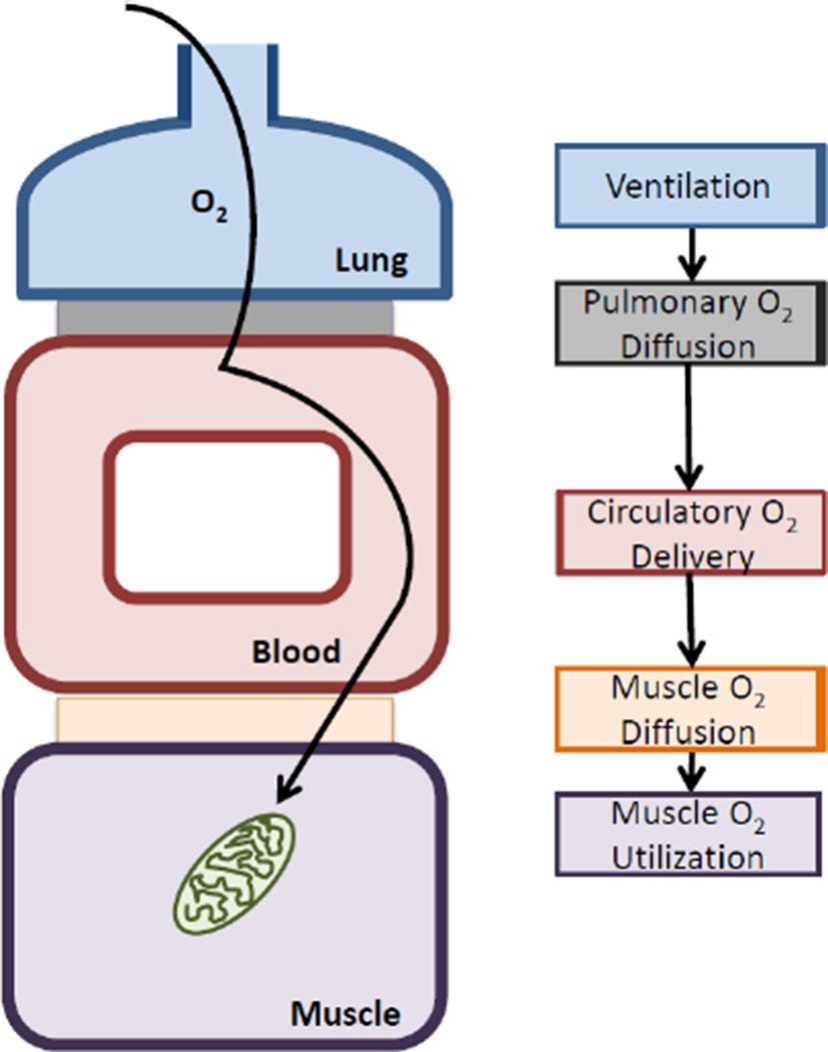
Schematic representation of the oxygen transport cascade. The features of the steps in the cascade associated with endurance exercise performance are well known, as is how these steps respond to training. The intermediate physiology is also well understood (e.g. the determinants of cardiac output). However, DNA-based explanations for the variability of key steps in the oxygen transport cascade have been hard to identify, and, as a result of physiological redundancy in adaptive responses, it is unclear whether the search for DNA-based explanations for the key elements of human performance outlined in the text will ever be able to tell a detailed deterministic story

Another way to think about endurance performance is via the so-called oxygen transport cascade (Fig. 1). In this cascade, the path of oxygen from the air to the tissues is considered. Therefore, in addition to cardiac output and red cell mass, factors such as the lung, capillaries, and skeletal muscle are incorporated into this approach. Using this schematic, it is possible to further summarize what is known about DNA-based explanations for differences in other key steps in the oxygen transport cascade.

### The Lung

A number of genome-wide association studies (GWAS) have been conducted in an effort to understand the role of DNA variants in lung function. The vast majority of these have focused on lung volumes, and there is little information on diffusing capacity. The take-home message from these studies is that there are a large number of potential common DNA variants that explain a tiny fraction of interindividual differences in lung function. Additionally, when so-called gene scores (composite values for a number of gene variants associated with a given phenotype) are constructed, lung function values in individuals in the highest quartile or quintile versus the lowest quartile or quintile frequently differ by only a few percentage points. These differences typically account for < 0.1 L of a given lung volume and are within the limits of the test–retest validity of spirometry [15]. Thus, there is no reason to believe that DNA variants explain any major difference in lung function in elite athletes, or their extremely high $\dot{V}{\text{O}}_{2\hbox{max} }$ values. Of note, individuals who have spent their entire life at high altitude have increased pulmonary diffusing capacity, but this is an adaptive response and is not intrinsic to populations who have lived at high altitude for generations [16].

### Cardiac Output and Stroke Volume

After the lung, the next step in the oxygen transport cascade is cardiac output. The right ventricle of the heart pumps blood through the lungs where it is oxygenated and returned to the left side of the heart, which delivers it to the systemic circulation. A hallmark of elite endurance performance is a high maximum cardiac output driven almost exclusively by a very large stroke volume [17]. To date, no DNA variants have been described that explain the impressive levels of stroke volume and cardiac output in elite athletes. Additionally, no DNA variants have been identified that explain why some people’s $\dot{V}{\text{O}}_{2\hbox{max} }$, and presumably cardiac output, increases more in response to exercise training than another’s. In the late 1990s and early 2000s, it was thought that differences in the ACE (angiotensin-converting enzyme) genotype might contribute to the high stroke volumes and $\dot{V}{\text{O}}_{2\hbox{max} }$ values seen in elite athletes, based on the potential for these variants to influence cardiac hypertrophy, but that seems unlikely at this time [18]. Additionally, the genetic contributions to maximum heart rate also appear physiologically trivial—only a few beats per minute [19].

### Red Cell Mass

In addition to cardiac output, red cell mass or total body hemoglobin are also important physiological determinants of $\dot{V}{\text{O}}_{2\hbox{max} }$. A high cardiac output that pumps anemic blood will not deliver much oxygen to the periphery. Thus, red cells and hemoglobin are required, together with a high cardiac output, to generate the impressive values seen in elite endurance athletes. At this time, there are no obvious genetic explanations for the high red cell masses seen in elite athletes, and these may be more generally linked to plasma volume expansion with exercise via the so-called critometer concept; in addition, there are examples of individuals with rare variants in their erythropoietin-related systems who have both high hematocrits and high values for $\dot{V}{\text{O}}_{2\hbox{max} }$ [20, 21].

### Peripheral Circulation

Once the blood leaves the left ventricle and enters the peripheral circulation it is delivered to the tissues. A key determinant of $\dot{V}{\text{O}}_{2\hbox{max} }$ is the ability to generate very high skeletal muscle blood flows. It is generally accepted that the capacity of skeletal muscle to vasodilate exceeds the ability (at least in humans) of the heart to sustain very high levels of blood flow in a large mass of active skeletal muscles, and also preserve blood pressure [22]. This is known as the ‘sleeping giant hypothesis’. Additionally, endurance exercise training does increase capillary density in the trained skeletal muscles, and there are also adaptations at the level of the resistance vessels and conducting vessels. As is the case for cardiac output and red cell mass, there is no clear DNA variant-based explanation for interindividual differences in these adaptations, or for the very high level of capillary density that can be seen in some highly trained individuals. It is also interesting to note that pharmacological blockade of vascular endothelial growth factor (VEGF) does not eliminate the vascular adaptations in animal models [23]. If at least some training-induced adaptations can occur when a key pathway is blocked, it seems unlikely that there might be a major impact of small effect size gene variants on these responses.

### Mitochondrial Density

One of the fundamental adaptations to endurance exercise training is the increase in mitochondrial density seen in trained skeletal muscle. When this was initially observed by John Holloszy in the mid-1960s, it was a revolutionary finding that initiated the era of exercise biochemistry [24–26]. Subsequent studies in humans showed that highly trained individuals with widely different $\dot{V}{\text{O}}_{2\hbox{max} }$ values have similar levels of mitochondrial adaptations in their skeletal muscles [6, 27]. As is the case for VEGF above, when knockout animals missing so-called ‘master regulators’ for mitochondrial biogenesis are trained, there are still significant mitochondrial adaptations [28]. Again, if at least some training-induced adaptations can occur when a key pathway is blocked or absent, it seems unlikely that there might be a major effect of small effect size gene variants on these responses.

**Fig. 2:**
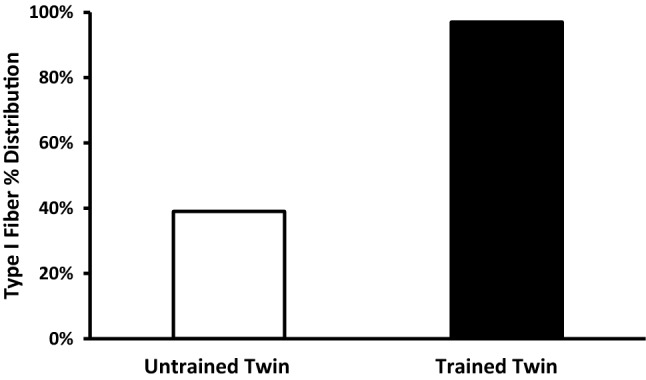
Marked differences in percentage slow-twitch fibers from the vastus lateralis of monozygotic twins aged in their mid-50s who were highly divergent for physical activity. The active twin had been engaged in competitive endurance training and competition for decades [29]

While twin studies show that skeletal muscle fiber type is highly heritable, there is ongoing discussion about so-called fiber-type transformation in humans in response to prolonged intense training [29–31]. In this context, a study in a unique set of identical twins highly divergent for physical activity over decades showed that muscle fiber type, especially for ‘slow twitch’ fibers, may be far more plastic than previously demonstrated (see Fig. 2) [32].

## Limitations and Potential Objections to This Perspective

There are a number of potential limitations to the perspectives outlined above. The most obvious is that very large cohorts of subjects (perhaps numbering in the hundreds of thousands) in conjunction with the phenotypes of interest and DNA sequence information are simply not available for the key steps in the oxygen transport cascade discussed in this review. For this sort of cohort to be a reality, beyond a blood test for genotyping, detailed measurements of gas exchange at rest and during submaximal and maximal exercise would be needed. Measurements of cardiac output and red cell mass would also be needed, as would serial measurements of blood lactate during graded exercise. Muscle biopsies to assess fiber type, mitochondrial function, and capillary density would also be essential. The financial and logistical barriers to such a research program seem formidable to say the least.

However, if such a cohort ever did emerge, it seems likely, based on the data from other phenotypes, that very large numbers of variants with very small effect sizes (relative risks of 1.1–1.5 are typically reported) would emerge [33]. Additionally, any rare DNA variants found in smaller case-control-like studies would likely show declining penetrance, and thus explain less of the physiology in any larger cohorts [34]. Importantly, the extent to which these variants would be causally or ‘casually’ associated with the physiological phenotype of interest would be uncertain, as would their overall explanatory power. To address these limitations in the studies of common disease risk, so-called polygenic gene scores have been developed [35]. However, the predictive utility of these scores is questionable for many complex phenotypes (e.g. obesity, diabetes, hypertension), and the overall genetic contribution to the phenotype of interest is much less than environmental and behavioral influences [36].

A final cautionary note is that for many complex human phenotypes, genetic association studies can have reproducibility issues, and also require diverse ethnic cohorts. The classic example of the reproducibility problem comes from studies of depression where a recent report found essentially no significant and reproducible genetic associations for depression [37].

## Conclusions

The above discussion of the oxygen transport cascade shows that while there is evidence, based on family and twin studies, for a genetic component of $\dot{V}{\text{O}}_{2\hbox{max} }$ and its trainability, it has been difficult to reconcile these observations with any specific large effect size gene variants or combinations of small effect size variants linked to key physiological pathways as a whole. Similar comments can be made about peripheral adaptations in skeletal muscle, and the determinants of efficiency are almost certainly complicated by biomechanical and skill-related factors as much as they are by genetic components. For considerations such as body size, similar observations can be made, and even in the case of ACTN3 variants associated with sprinting or power performance, the effect sizes are tiny and there are examples of elites with the ‘wrong’ genotype [38, 39]. Additionally, in some sports such as swimming, the ACTN3 genotype does not clearly segregate in sprinters versus endurance athletes [40].

The obvious question is why? One emerging concept is that there are many potential genetic pathways to a given phenotype [41]. This concept is consistent with ideas that biological redundancy underpins complex multiscale physiological responses and adaptations in humans [42]. From an applied perspective, the ideas discussed in this review suggest that talent identification on the basis of DNA testing is likely to be of limited value, and that field testing, which is essentially a higher order ‘bioassay’, is likely to remain a key element of talent identification in both the near and foreseeable future [43]. While it is possible that more explanatory DNA-based associations for complex exercise-related traits might emerge if detailed physiological phenotyping of large cohorts of humans is performed, there are many limitations to this perspective. In this context, the advocates of ever-bigger Ns should carefully review the limits of this approach from studies of other complex phenotypes as they make the case for a ‘more is better’ approach to future studies.
